# Racial and ethnic disparities in fatal police shootings: Variation across U.S. states and the role of firearm ownership

**DOI:** 10.1371/journal.pone.0333424

**Published:** 2026-03-11

**Authors:** Roland Neil, Shawn Bushway, Terry L. Schell, Andrew R. Morral, Rosanna Smart

**Affiliations:** 1 RAND, Santa Monica, California, United States of America; 2 Department of Public Administration & Policy, University at Albany, SUNY, Albany, New York, United States of America; Iowa State University, UNITED STATES OF AMERICA

## Abstract

Fatal police shooting rates vary greatly across U.S. states, and states with higher firearm ownership rates tend to have higher rates of police shootings. Yet, despite well-documented national racial and ethnic disparities in fatal police shootings, prior research has not established how much these disparities vary across states, nor whether firearm ownership rates are associated with racial and ethnic disparities in police shootings. This article characterizes state-level fatal police shooting rates and racial/ethnic disparities in those rates, and tests whether firearm ownership rates are associated with these outcomes. Using 2015–2020 data on police shootings from the *Washington Post,* we predict fatal police shooting rates by state, year, and race/ethnicity using Bayesian multilevel count models. The degree of Black-White disparities in fatal police shooting rates varies by an order of magnitude across states, although Black rates exceed White rates in every state. In contrast, though Hispanic rates exceed White rates nationally, the opposite is true for 31 states. Southwestern states with large Hispanic-White disparities, relatively high shooting rates, and large Hispanic populations contribute most to the observed national disparity. Fatal police shooting rates are strongly associated with firearm ownership rates, but racial/ethnic disparities are either not related to firearm ownership rates or are larger in states with lower firearm ownership rates, depending on the group comparison and disparity metric. While state-level variation in firearm ownership may impact fatal police shooting rates, it is unlikely that higher firearm ownership rates explain why some states have such large racial/ethnic disparities compared to others.

## Introduction

Police officers fatally shoot about 1,000 people each year in the United States, for a fatality rate—0.3 per 100,000—that far exceeds that of other wealthy democracies [1]. This burden falls disproportionality on racial minorities. Nationally, Black individuals face a probability of death by police shooting approximately twofold higher than Hispanic individuals and threefold higher than White individuals [2]. The high and disparate rates of fatal police shootings in America are a source of concern for researchers, policymakers, and the public [3].

Though fatal police shootings are a national challenge, there is pronounced state-level variation; rates vary more than tenfold across states [4]. Relatedly, national racial/ethnic disparities in fatal police shootings may mask heterogeneity across states in the degree—or direction—of disparities. For instance, these might arise due to state differences in the specific circumstances of racial/ethnic groups (e.g., in socioeconomic status, immigration levels, segregation patterns, and other historical legacies facing groups that vary by state); in the police who respond to encounters (e.g., due to differences in police culture); and in the ecological contexts in which encounters occur (e.g., due to state differences in urbanicity). However, since little research has examined racial/ethnic-specific fatal police shootings at the state level [5,6], the extent to which disparities in police shootings vary by state remains an open question.

An emerging strand of research focuses on the extent to which state differences in police shooting rates are explained by variation in firearm availability, ownership, or policies [4–11]. These studies generally find that states’ overall fatal police shooting rates are positively associated with firearm availability and with more permissive firearm laws. This association may reflect that officers perceive greater risks where firearms are more common, increasing the likelihood that they will use deadly force. Because such risk perception is prospective, it may also impact encounters in which civilians turned out to be unarmed [4,5].

The relationship between state-level firearm ownership and fatal police shootings may vary by race through two pathways. First, while racial stereotypes can influence perceived danger [12], the prevalence of firearms might interact with these stereotypes to make them either more or less important in impacting officers’ use of deadly force. Greater firearm availability might increase racial disparities if officers perceived (due to stereotypes) that the increased risks from firearms was primarily from certain minority groups. Conversely, since firearm ownership is more prevalent among White households [13], and the presence of firearms increases the likelihood of fatal police encounters, disparities might be smaller in states with more firearms. Put differently, the presence of firearms may lead officers to downweight racial stereotypes. Second, firearm availability might impact officers’ contextual cues of danger at more local scales [14], for instance, it might exacerbate neighborhood differences in perceived danger. If this maps onto patterns of racial segregation, it could disproportionately increase minority individuals’ (especially Black individuals’) exposure to officers with elevated perceptions of danger. It is also possible that the risk of fatal police shooting increases similarly across racial/ethnic groups in states with higher firearm ownership rates, especially given that the only prior study examining a similar question found no statistically significant Black versus non-Black differences in how a proxy of state-level firearm availability related to fatal police shootings rates [5].

In this article, we use 2015–2020 police shooting data from *The Washington Post* in a two-part analysis to study state-level differences in racial/ethnic disparities in fatal police shooting rates. First, we characterize state-level variability in fatal police shootings rates and racial/ethnic disparities (Black-White and Hispanic-White). This analysis establishes the extent to which states vary in the size and direction of their racial/ethnic disparities, and the relationship between disparities and rates of fatal police shootings at the state level. Second, we model the relationships between state-level firearm ownership rates and race/ethnicity-specific fatal police shooting rates. Like prior work, this analysis enables an examination of the association between firearm ownership rates and fatal police shootings rates. However, it builds on that research by examining whether state-level differences in firearm ownership rates are associated with racial/ethnic disparities in fatal police shootings.

Together, these analyses contribute to scholarship on police shootings in several ways. For one, much prior research focuses on racial/ethnic disparities at the individual, neighborhood, city, and county levels, without characterizing state differences [15–28]. We establish that states, though often overlooked, are a key axis of variation for racial/ethnic disparities in fatal police shootings. Not only do national racial/ethnic disparities in fatal police shooting mask important heterogeneity across states, but as we discuss, state heterogeneity in police shooting rates and disparities imply that some states play an outsized role in contributing to the aggregated, national patterns of disparities. We also build on prior work to more fully describe the association of state firearm ownership rates and racial/ethnic disparities in fatal police shootings, finding that higher firearm ownership rates are associated with higher fatal police shooting rates overall but not with larger racial/ethnic disparities in those deaths. Ultimately, our results point to state differences as an underappreciated dimension of racial/ethnic disparities in police shootings, one that may point towards effective policy solutions to address such disparities.

## Methods

### Data

Our outcome measure is *fatal police shootings*, constructed with data from *The Washington Post’*s Fatal Force database for the years 2015–2020 [29]. During this period, the database was built through extensive sourcing of incidents from media and police reports coupled with independent verification. Of the 5,944 unique fatal police shootings in the 50 states, our analysis focuses on the 1,430 (non-Hispanic) Black, 2,770 (non-Hispanic) White, and 1,021 Hispanic deaths (total = 5,221), as there are not enough fatal shootings of other racial/ethnic groups for precise estimates. Of the 5,944 fatalities, 473 (8%) are missing decedent race/ethnicity. These are omitted from the main analysis but were included with imputation in supplementary analyses; results were substantively unchanged (S1 Table). We aggregate fatal shooting counts to the state-year-race/ethnicity level. Corresponding *population* data for calculating shooting rates were drawn from U.S. Census Bureau single-race population estimates, accessed through CDC WONDER [30]. We measure *household firearm ownership rates* at the state level using RAND’s estimates for the years 2015–2018 [31]. All measures were combined into a state-year-race/ethnicity-level analytic dataset, yielding 900 observations (50 states * 6 years * 3 racial/ethnic groups).

### Models

We fit two multilevel Bayesian models. Model 1 is a random slope model used to characterize state-level differences in fatal police shooting levels and racial/ethnic disparities. Inspired by Gelman et al.’s [32] analysis of racial/ethnic disparities in pedestrian stops, the model takes the form:


$$Y_{rst}\sim NB\left(\lambda_{rst},\;\varphi \right),$$ (1)

$$\text{log}\;\left(\lambda_{rst}\right)=\;\beta_{\text{0}}+\beta_{\text{1}s}+\beta_{\text{2}t}+\beta_{\text{3}}*Black+\beta_{\text{4}}*Hispanic\;{\;+\;\beta }_{\text{5}s}*Black+\beta_{\text{6}s}*Hispanic+\text{log}\left(p_{rst}\right)\;,$$

$$[\begin{array}{c} \beta_{\text{1}s}\\ \beta_{\text{5}s}\\ \beta_{\text{6}s} \end{array}]\sim MVN\left([\begin{array}{c} \text{0}\\ \text{0}\\ \text{0} \end{array}]\;,𝛴\right)\;,$$

$$\beta_{\text{2}t}\sim N\left(\text{0},\sigma^{\text{2}}\right),$$

where the number of fatal police shootings $Y_{rst}$ for racial/ethnic group *r* in state *s* and year *t* follows a negative binomial distribution with expected value $\lambda_{rst}$ and overdispersion parameter φ. The log of $\lambda_{rst}$ is modeled as a grand intercept $\beta_{\text{0}}$, plus state and year random intercepts $\beta_{\text{1}s}$ and $\beta_{\text{2}t}$, fixed effects $\beta_{\text{3}}$ and $\beta_{\text{4}}$ for the Black and Hispanic groups (White is the reference category), and state-specific random slopes $\beta_{\text{5}s}$ and $\beta_{\text{6}s}\;$for the Black and Hispanic groups, respectively. The fixed effects $\beta_{\text{3}}$ and $\beta_{\text{4}}$ capture the average racial disparities across states, where the random slopes $\beta_{\text{5}s}$ and $\beta_{\text{6}s}$ capture how each state’s racial disparities deviate from those average disparities. The log of population p is included as an offset.

Model 2 examines how levels and disparities in fatal police shooting rates vary across states with different firearm ownership rates. It builds on Model 1 by adding state household firearm ownership rates as a predictor. This model has the form:


$$Y_{rst}\;\sim \;NB\left(\lambda_{rst},\;\varphi \right),$$ (2)

$$\begin{array}{cc} \text{log}\left(\lambda_{rst}\right)\;= & \;\beta_{\text{0}}+\beta_{\text{1}s}+\beta_{\text{2}t}+\beta_{\text{3}}*Black+\beta_{\text{4}}*Hispanic\\ \;\;\; & +\;\beta_{\text{5}s}*Black+\beta_{\text{6}s}*Hispanic\\ \;\;\; & +\beta_{\text{7}}*F+\beta_{\text{8}}*F*Black+\beta_{\text{9}}*F*Hispanic+\text{log}\left(p_{rst}\right)\;, \end{array}$$

$$[\begin{array}{c} \beta_{\text{1}s}\\ \beta_{\text{5}s}\\ \beta_{\text{6}s} \end{array}]\;\sim \;MVN\left([\begin{array}{c} \text{0}\\ \text{0}\\ \text{0} \end{array}],\;\Sigma \right),$$

$$\beta_{\text{2}t}\;\sim \;N\left(\text{0},\;\sigma^{\text{2}}\right),$$

in which state firearm ownership rate F influences the expected number of shootings through $\beta_{\text{7}}$, and through race/ethnicity-specific interaction terms with Black and Hispanic, corresponding to coefficients $\beta_{\text{8}}$ and $\beta_{\text{9}}$.

The models were fit using Hamiltonian Monte Carlo (HMC), implemented in R using the brms package, which interfaces with Stan [33,34]. Examinations of Monte Carlo standard errors, $\hat{R}$ statistics, effective sample size, and trace plots showed that the models converged properly. Posterior predictive checks revealed that both models fit the data well (S2 Table). All models have weakly informative Bayesian priors. Specifically, the priors are: Normal(0, 10^2^) for the intercept, Normal(0, 5^2^) for other fixed effects, half-*t*(df = 3, location = 0, scale = 2.5) for the standard devia*t*ions of the random effects, LKJ(1) for the correlations between the state-level random terms, and Exp(0.02) for the (reciprocal) dispersion parameter.

## Results

### National fatal shooting rates and disparities

Table 1 presents national fatal police shooting rates per 100,000 over the 6-year study period observed in our data by race/ethnicity, as well as the risk ratios and risk differences for Black and Hispanic individuals relative to White individuals. Nationally, the Black fatal police shooting rate is 3.54 whereas the White rate is 1.40, a rate difference of 2.14. The Black-White risk ratio is 2.52, meaning that the Black rate is 152% higher than the White rate. The national Hispanic rate of 1.74 is also higher than the White rate. The rate ratio of 1.24 indicates that the Hispanic rate is 24% higher than the White rate. As expected, our model-based estimates closely resemble the observed data (S3 Table). S4 Table contains regression output for Models 1 and 2 (and S5 Table demonstrates these results are robust to a Poisson specification).

**Table 1:** Fatal police shooting rates by race and national disparities.

Race/Ethnicity	Fatal Police Shooting Rate	Risk Difference Compared to White Rate	Risk Ratio Compared to White Rate
White	1.40	N/A	N/A
Black	3.54	2.14	2.52
Hispanic	1.74	0.33	1.24

Fatal police shooting rates are per 100,000 residents over the 6-year study period.

### Characterizing state-level differences in fatal police shootings

**Fig 1:**
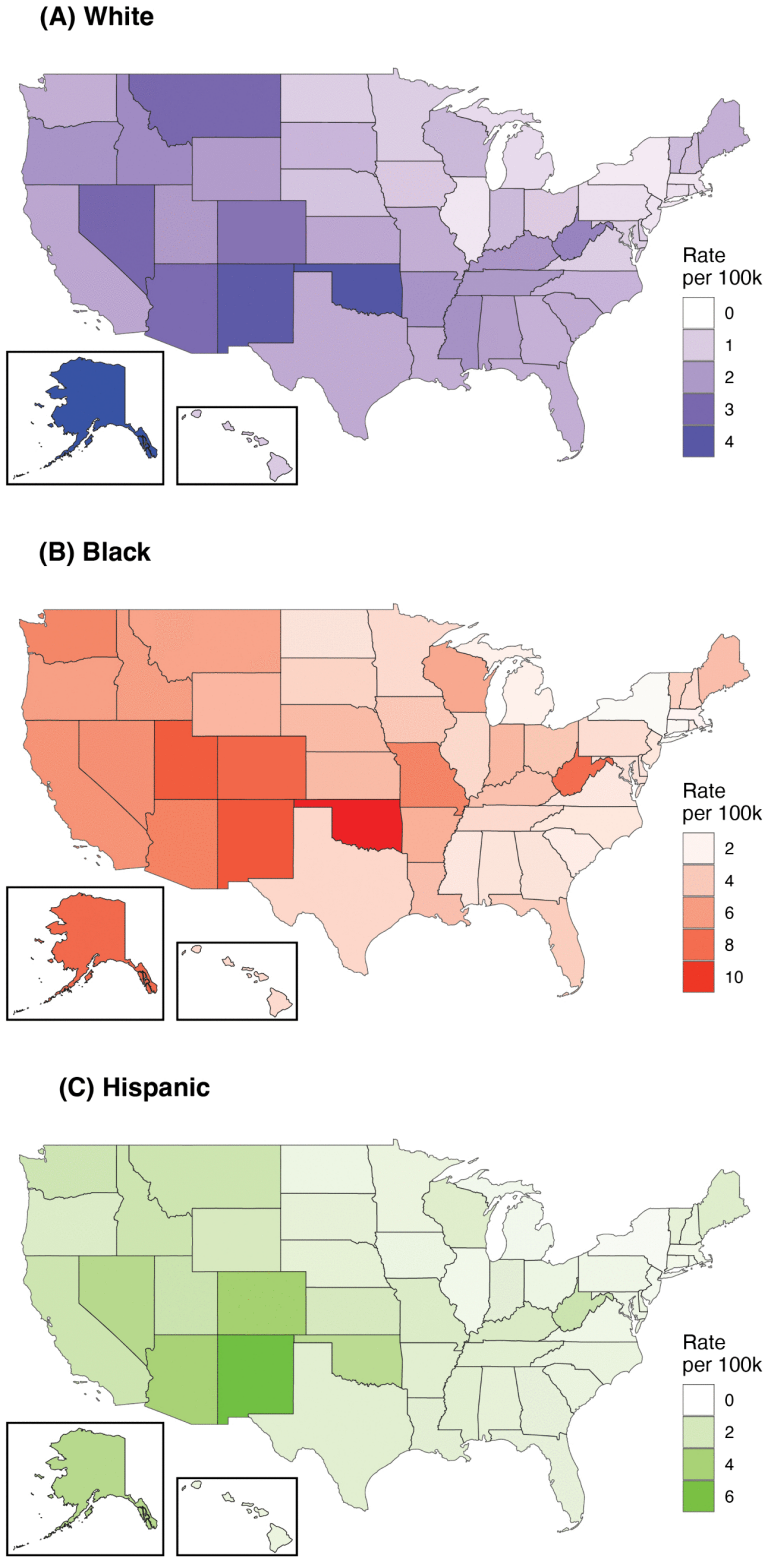
Fatal police shooting rates per 100,000 residents over the 6-year study period. These modeled state-level rates are for (A) White, (B) Black, and (C) Hispanic individuals. Alaska and Hawaii are not to scale. Maps created using data from the U.S. Census Bureau.

Drawing on Model 1, Fig 1 maps fatal police shooting rates by state and race/ethnicity (rates are per 100,000 residents over the 6-year study period). There is regionality to these rates for all groups: the highest rates tend to be in the West, the lowest in the Northeast, and the South generally falls in between these extremes. White fatal police shooting rates vary elevenfold across states, ranging from 0.39 in New York (80% Bayesian credible interval [CrI]: 0.32, 0.47) to 4.53 in Alaska (CrI: 3.43, 5.90). Black rates vary by a factor of six, from a low of 1.75 in New York (CrI: 1.46, 2.08) to 10.47 in Oklahoma (CrI: 8.33, 13.05). Hispanic rates vary twentyfold across states, from 0.31 in New York (CrI: 0.22, 0.41) to 6.28 in New Mexico (CrI: 5.31, 7.39).

**Fig 2:**
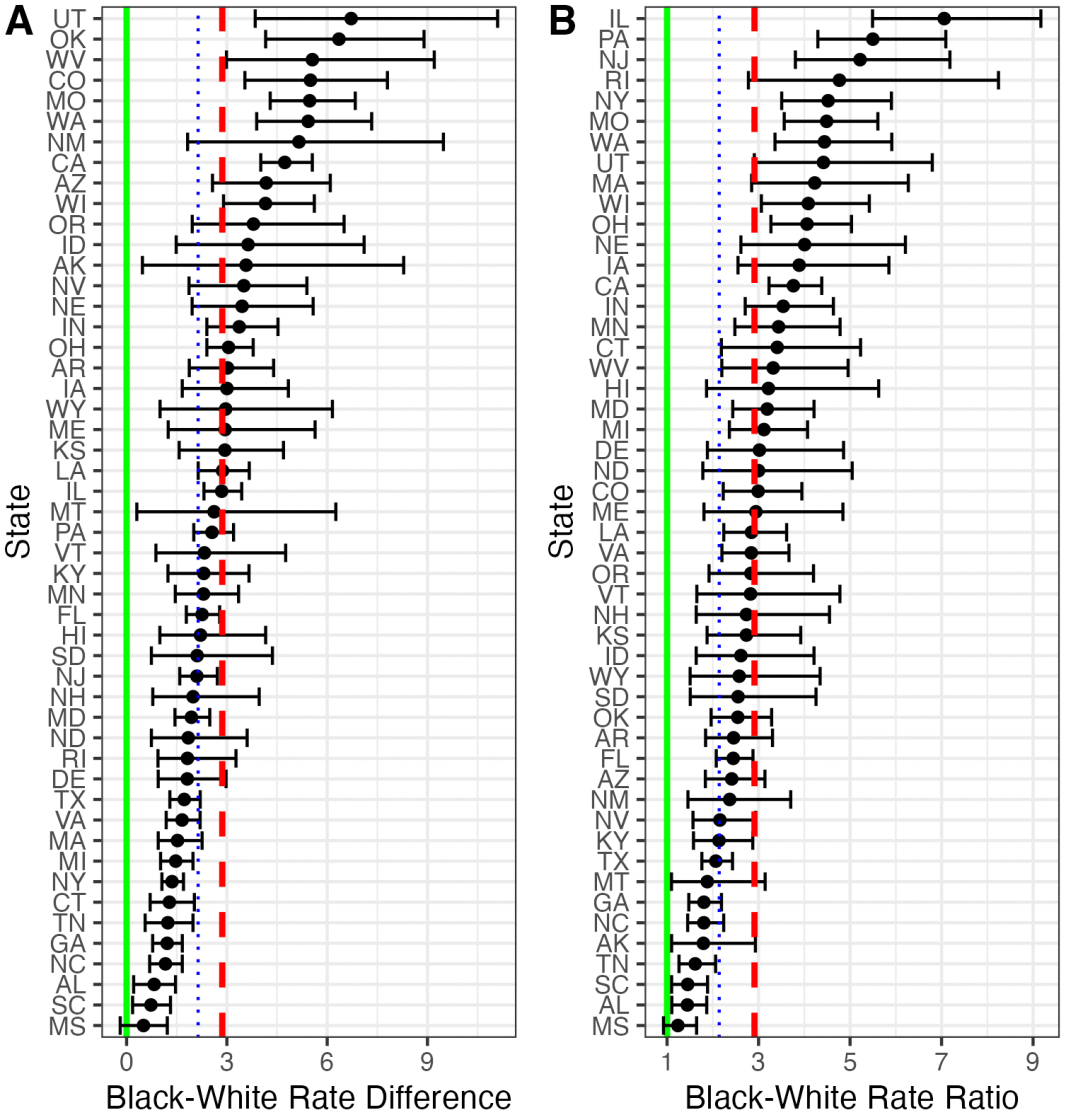
State-specific modeled Black-White disparities. Expressed as (A) rate differences and (B) rate ratios. Rates are per 100,000 residents over the 6-year study period. The red dashed lines indicate the cross-state average, the blue dotted lines the national average, and the green solid lines represent Black-White parity for the respective metrics. Points indicate posterior medians; bars indicate 80% credible intervals.

Fig 2 presents the modeled disparities between fatal police shooting rates of Black individuals compared to White individuals. In every state, police fatally shoot Black individuals at higher rates than White individuals, but the magnitude of Black-White rate differences (Fig 2A) ranges from 0.50 in Mississippi (MS) (CrI: −0.19, 1.22) to 6.72 in Utah (UT) (CrI: 3.85, 11.10). In much of the South (e.g., Mississippi, South Carolina [SC], Alabama [AL], North Carolina [NC,] Georgia [GA], and Tennessee [TN]), Black-White rate differences are comparatively small. In contrast, Western states including the Southwest (e.g., Utah, New Mexico [NM], Colorado [CO], Washington [WA], California [CA]), have especially large Black-White rate differences, as do Oklahoma (OK), West Virginia (WV), and Missouri (MO).

Measuring Black-White disparities as rate ratios instead of rate differences yields a slightly different pattern (Fig 2B). There is a positive correlation between the state-level rate ratios and rate differences presented in Fig 2 (r = 0.44, CrI: 0.30, 0.58). As with rate differences, rate ratios tend to be smallest in the South. The starkest divergence is that many of the states with comparatively small rate differences have the highest rate ratios (e.g., New York [NY], Rhode Island [RI], Massachusetts [MA], New Jersey [NJ]). This reflects that states with lower White fatal police shooting rates have larger Black-White rate ratios even though they have smaller Black-White rate differences in fatal police shootings. Indeed, Black-White rate differences are positively correlated with White fatal police shooting rates (r = 0.38, CrI: 0.18, 0.56), meaning that states with higher White shooting rates tend to have Black rates that exceed those Whites rates by a larger amount than average (see S1 Fig for scatterplots). In contrast, the association between White fatal police shootings and Black-White rate ratios is negative (r = −0.48, CrI: −0.58, −0.36) (S2 Fig). Relatively small difference between Black and White fatal police shooting rates are larger on the rate ratio scale when White rates are close to zero.

**Fig 3:**
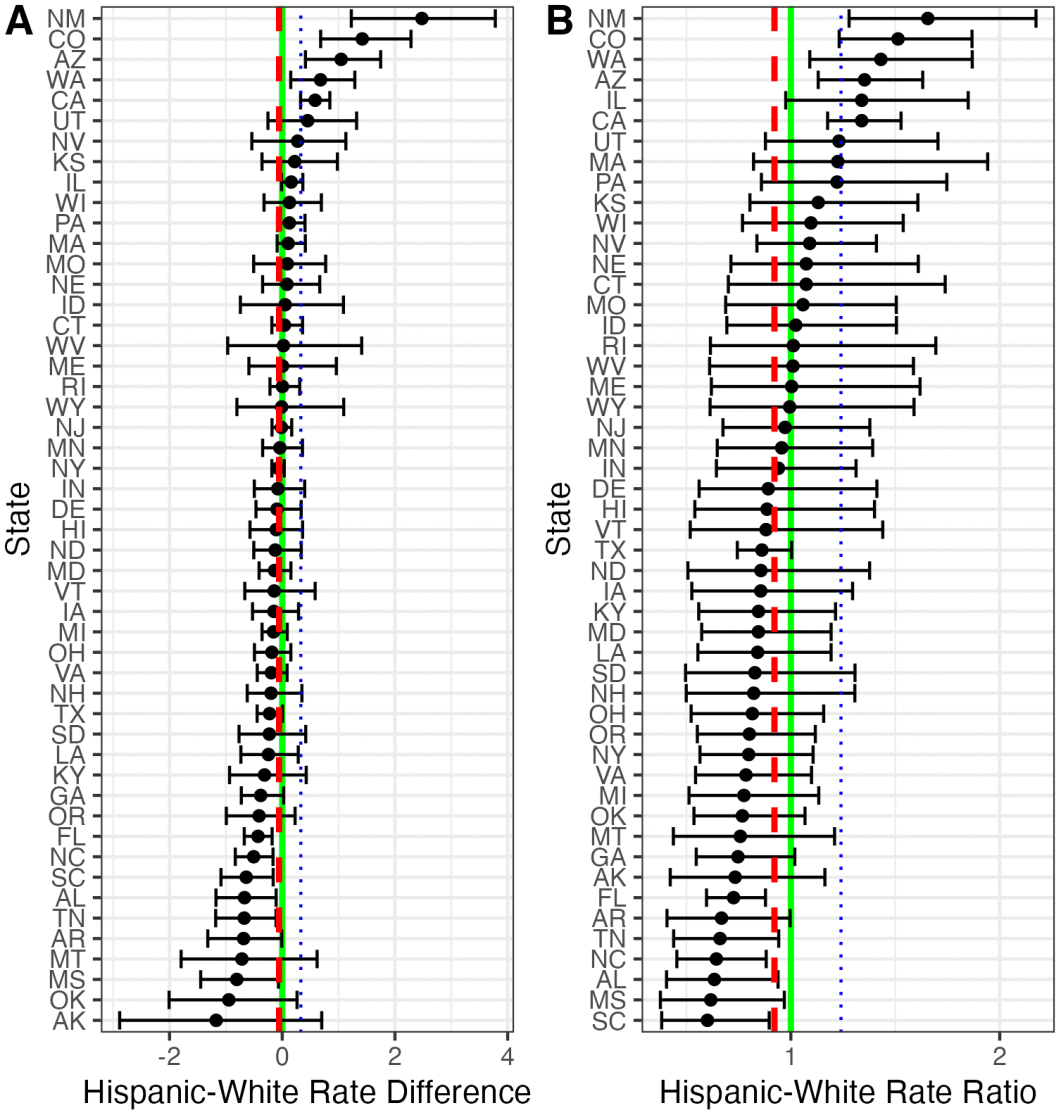
State-specific modeled Hispanic-White disparities. Expressed as (A) rate differences and (B) rate ratios. Rates are per 100,000 residents over the 6-year study period. The red dashed lines indicate the cross-state average, the blue dotted lines the national average, and the green solid lines represent Hispanic-White parity for the respective metrics. Points indicate posterior medians; bars indicate 80% credible intervals.

In 31 states, the medians of the posterior Hispanic-White rate differences are negative, indicating that fatal police shooting rates are lower for Hispanic than White individuals in most states (Fig 3A). This is despite the fact that, nationally, Hispanic fatal police shooting rates exceed White rates (represented by the blue line in Fig 3A). The states where White rates exceed Hispanic rates by the most tend to be in the South. The largest positive Hispanic-White rate differences, where Hispanic rates exceed White rates, are concentrated in Western and Southwestern states (e.g., New Mexico, Colorado, Arizona [AZ], Washington, California). The rate ratio version of this analysis reveals a similar pattern of state differences (Fig 3B). Reflecting this, the Hispanic-White rate differences shown in Fig 3A are strongly correlated with the rate ratios shown in Fig 3B (r = 0.87, CrI: 0.80, 0.91).

There is little association between White shooting rates and Hispanic-White rate differences (r = −0.01, CrI: −0.26, 0.26). However, some of the states with the highest White fatal police shooting rates and largest positive Hispanic-White rate differences (New Mexico, Arizona, Nevada [NV], Colorado and California) are also states with especially large Hispanic populations (S1 Fig). Finally, there is a positive relationship (r = 0.44, CrI: 0.08, 0.75) between Black-White and Hispanic-White rate differences, meaning that states with large rate differences for one of these minority groups tend to have large rate differences for the other group too. Examining these two associations using rate ratios yields the same general pattern of results (S1 Fig).

### Associations with firearm ownership rates

**Fig 4:**
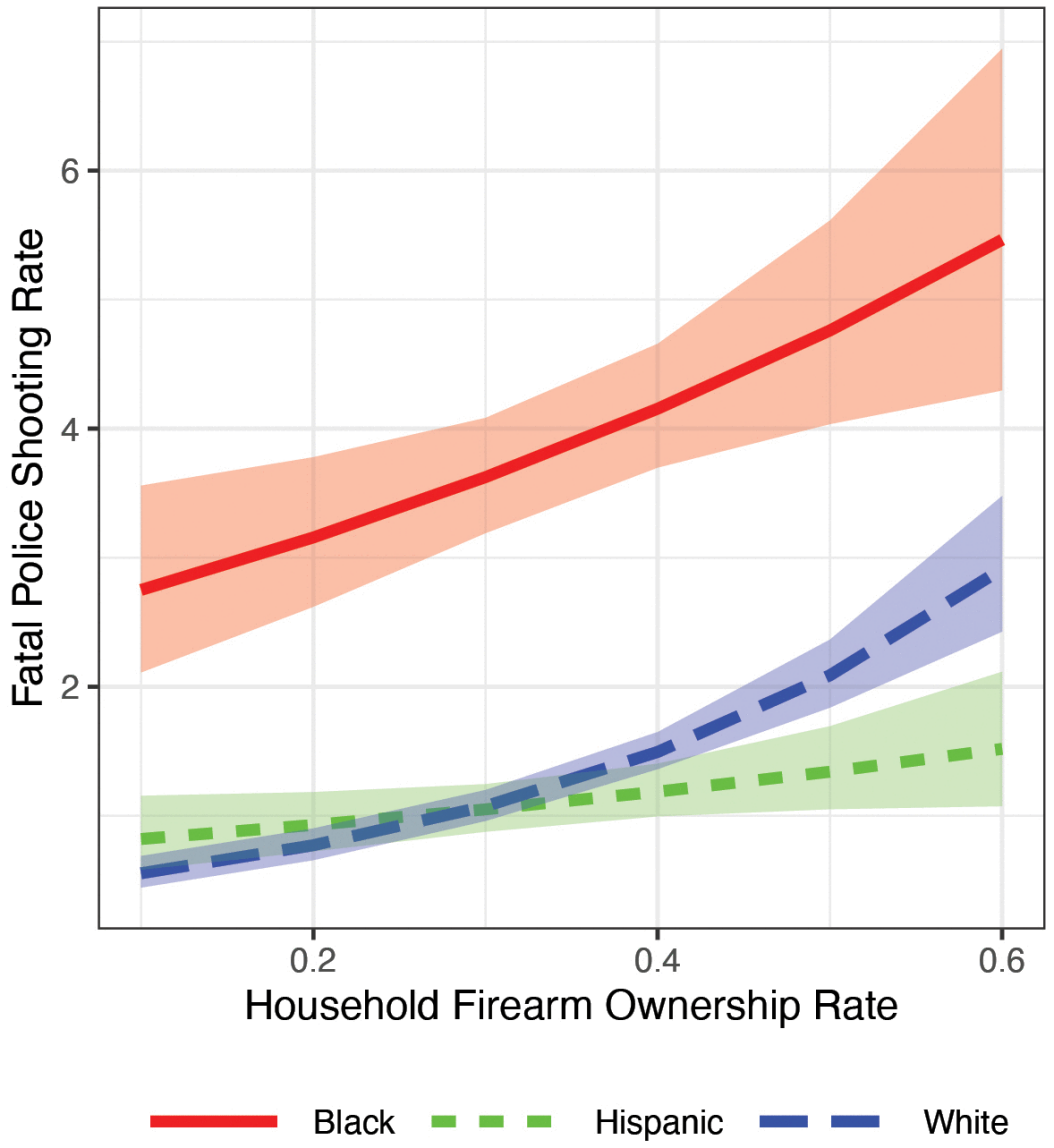
Race/ethnicity-state-level associations between police shooting deaths and household firearm ownership rates. Ribbons indicate 80% credible intervals. Rates are expressed as the number of police shootings per 100,000 over the 6-year study period.

Using Model 2, we find that states with higher firearm ownership rates tend to have higher fatal police shooting rates for all racial/ethnic groups (Fig 4), but the gradient of this relationship differs by race/ethnicity, which has implications for disparities. The large Black-White differences in rates are relatively stable across the range of firearm ownership rates. Rates differ by between 2.27 (CrI: 1.65, 2.94) in states with 12% ownership rates (which is the average rate for the bottom decile that includes Hawaii, Massachusetts, New Jersey, New York, and Rhode Island) and 2.64 (CrI: 1.54, 3.86) in those with 59.5% ownership rates (which is the average for the top decile that includes Alaska, Montana, South Dakota, West Virginia, Wyoming). The difference between those values is 0.37 (CrI: −1.20, 2.02); thus, although the two lines are approximately parallel, the data do not definitively rule out some interaction on the rate difference scale. However, there is strong evidence of an interaction on the rate ratio scale. White fatal police shooting rates approach zero at the lowest levels of ownership, a small fraction of the rates for White individuals in high ownership states, whereas rates for Black individuals do not change by a similarly large proportion across levels of firearm ownership. Consequently, Black-White rate ratios for police shootings are much higher in states with lower firearm ownership levels. Specifically, the rate ratio is 4.86 (CrI: 3.78, 5.98) among states with 12.5% ownership as compared to 1.93 (CrI: 1.53, 2.37) among those with 59.5% ownership (a difference of 2.93 [CrI: 1.53, 4.33]).

As can be seen in Fig 4, the size and directionality of Hispanic-White disparities vary by firearm ownership rate, with White fatality rates being higher than Hispanic rates in states with higher rates of firearm ownership but lower in states with lower ownership rates. For instance, the estimated fatal police shooting rate in states where 12% of households own firearms is 0.27 (CrI: 0.07, 0.50) higher for Hispanic than White individuals, compared to 1.34 (CrI: 0.86, 1.80) higher for Whites than Hispanics in states where 59.5% of households own firearms. The difference between those differences is 1.61 (CrI: 1.02, 2.19). Similarly, the rate ratio is 1.45 (CrI 1.12, 1.81) in states with 12% ownership (i.e., Hispanic fatality rates are 44% higher than White rates), compared to 0.54 (CrI: 0.40, 0.69) in states with 59.5% ownership (a difference of 0.91 [CrI: 0.49, 1.37]).

S3 Fig demonstrates these results are robust to a quadratic specification. S4 Fig plots Black-White and Hispanic-White rate ratios and rate differences as a function of firearm ownership rates, drawing on the same model as Fig 4. S5 Fig is like Fig 4 but uses state-level firearm ownership rate data separated by race/ethnicity [35]. Although the racial/ethnic groupings differ in that analysis (non-Hispanic White versus Hispanic and Black), the patterns are consistent with Fig 4.

## Discussion

National patterns of fatal police shootings mask large state-level heterogeneity in both levels and disparities. The rate of fatal police shootings varies dramatically across states, by a factor of about 11 for White individuals, 6 for Black individuals, and 20 for Hispanic individuals. There is pronounced regionality, with Western states tending to have the highest rates and Northeastern states the lowest. Black fatal police shooting rates exceed White rates in every state, but Black-White differences vary greatly across states, tending to be smallest in the South and largest in Western and Southwestern states. States with higher White fatal police shooting rates tend to have larger Black-White rate differences. In contrast, there is a negative association between White fatal police shooting rates and Black-White rates ratios. Relative or proportional differences between Black and White fatality rates are greatest in states with the lowest White rates, which are the same states where absolute differences between Black and White rates tend to be smallest.

In contrast to those Black-White differences, Hispanic fatal police shooting rates are lower than White rates in 31 states, even though nationally Hispanic rates are 24% higher than White rates. In further contrast, state patterns of Hispanic-White disparities are consistent regardless of whether measured using rate differences or rate ratios. In particular, the states where rate ratios are smallest and Hispanic rates are below White rates by the most in absolute terms tend to be in the South, whereas the states where the opposite pattern occurs—those where Hispanic rates exceed White rates by the most—tend to be in the West. Also, although Hispanic-White disparities are barely related to states’ fatal police shooting levels, many of those Western states (e.g., Arizona, New Mexico, Colorado) have high fatal shooting rates of White individuals. Although Hispanic-White and Black-White patterns exhibit notable differences across states, they are nonetheless modestly positively correlated.

These results suggest that a few states are disproportionately responsible for the fact that Hispanic fatal police shooting rates exceed White rates nationally. It would be mistaken to examine the degree of disparities alone in trying to assess how states contribute to the national average, since racial/ethnic-specific population sizes and fatal police shooting rates also matter. Though, we found Southwestern states have some of the largest disparities and high fatal police shooting rates, and these are states with large Hispanic populations. Confirming this intuition, Hispanic fatal police shooting rates are 7% lower than White rates when excluding California, Arizona, and New Mexico, as opposed to 24% higher when including them. As such, to explain the national pattern of Hispanic-White disparities in fatal police shootings, a focus on the Southwest is essential. While the heterogeneity in Black-White disparities means some states influence the national average in part by having larger disparities than others, the national average reflects a story that plays out within every state: Black fatality rates exceed White rates.

In modeling firearm ownership rates and race-specific fatal police shootings, we found that states with higher rates of firearm ownership tend to have smaller Hispanic-White disparities in fatal police shootings, with Hispanic rates often being lower than White rates. For Black-White patterns, we found that Black-White rate differences are relatively consistent across states characterized by different firearm ownership rates, although there was some imprecision in that estimate. Black-White rate ratios, however, are much larger in states with lower firearm ownership rates.

Notably, none of these patterns suggest disparities tend to be larger in states with higher firearm ownership rates. As such, policies that aim to reduce levels of police shootings through curtailing firearm availability are unlikely to reduce racial/ethnic disparities. Yet, the number of people killed of a given race/ethnicity is not only a function of disparities but also of the overall fatal police shooting levels. It is thus consequential that we find that lower firearm ownership rates are associated with markedly lower fatal police shootings rates for all three racial/ethnic groups, consistent with prior work that focuses on overall rates [4–11]. Our work, like most prior work on this topic, is descriptive and associational, meaning the causal impact of changes to firearm ownership on fatal police shootings remains unclear. Even if lower firearm ownership rates would causally reduce fatal police shooting rates, however, our findings cast doubt on whether that would ameliorate the large Black-White disparity in the probability of being fatally shot by police. After all, in the states with the lowest levels of gun ownership, the risk of being fatally shot by police for Black individuals is an even larger multiple of the risk for White individuals.

Our findings might not generalize to police shootings that resulted in non-fatal injuries, which are roughly as common as fatal shootings [36,37]. The fraction of police shootings that are lethal (i.e., the case fatality rate) is lower for non-Hispanic Black individuals compared to non-Hispanic White or Hispanic individuals [20,36], meaning that Black-White disparities for all police shootings are larger than those we report. Also, case fatality rates vary across states [38]; it is unclear the extent to which that reflects random variation, but it could be caused by many differences between states, for example, such as in the accessibility of trauma care [5,37]. This prior research suggests our results would likely vary if studying all police shootings, though we lack the non-fatal police shooting data to empirically establish how so or by how much, making this an interesting direction for future research.

Our analyses do not estimate causal treatment effects. Omitted third factors may explain both rates of firearm ownership and police shooting rates, meaning our estimates of the association of ownership and shooting rates are larger than the true effects of ownership on shootings, or vice versa. However, our conclusion that lower firearm ownership rates are unlikely to lower disparities in fatal police shootings is based on our findings of there being no associations or associations that run in the opposite direction. While it is possible for associations to be masked by omitted third variables, we judge that to be unlikely on the scale needed to overturn this conclusion, though this judgement is a possible source of error, again pointing to the value of causal methods for future work in this area. Relatedly, there are many different possible causal explanations for our descriptive findings, such as differences in the rate at which police officers are placed into potentially life-threatening scenarios across states or differences in police training or culture. Understanding the causal mechanisms underlying our results is both an interesting direction for future work and one that could carry great practical relevance in addressing fatal police shooting levels and disparities.

This study has established that the national pattern of racial/ethnic disparities masks large heterogeneity across states. While Black fatal police shooting rates are higher than White rates in every state, the size of those differences varies by an order of magnitude. This a major type of variation that has gone largely overlooked but, if better understood, might help in addressing Black-White disparities in fatal police shootings. Efforts to reduce Hispanic-White disparities should focus on understanding why those disparities are so pronounced, and the rate of fatal police shootings are so high, in Southwestern states. Also, this study adds to the mounting associational evidence that state-level firearm ownership might be a relevant pathway for reducing fatal police shootings in America. However, interventions focusing on that should not be expected to reduce racial or ethnic disparities in those fatalities. Counterintuitively, policies that leave common metrics of racial or ethnic disparities unchanged, or even exacerbate them, may be effective solutions in reducing the harms faced by racialized minority groups.

## Supporting information

S1 Table Output for negative binomial models predicting fatal police shootings using imputed data. Coefficients are expressed as log rate ratios, brackets indicate 80% Bayesian credible intervals. Both models also contain the log of population as an offset, random intercepts for year and state, and state-race/ethnicity random slopes. Cf. S4 Table, which contains the regression output for the models used in the main results. N = 900.
(PDF)

S2 Table Posterior predictive checks. (PDF)

S3 Table Model-based fatal police shooting rates by race and national disparities. Fatal police shooting rates are per 100,000 residents over the 6-year study period. 80% Bayesian credible intervals are in brackets. Cf. Table 1, which contains the analogous quantities using the observed data as opposed to these model-based estimates.
(PDF)

S4 Table Output for negative binomial models predicting fatal police shootings. Coefficients are expressed as log rate ratios, brackets indicate 80% Bayesian credible intervals. Both models also contain the log of population as an offset, random intercepts for year and state, and state-race/ethnicity random slopes. N = 900.
(PDF)

S5 Table Output for Poisson models predicting fatal police shootings. Coefficients are expressed as log rate ratios, brackets indicate 80% Bayesian credible intervals. Both models also contain the log of population as an offset, random intercepts for year and state, and state-race/ethnicity random slopes. N = 900.
(PDF)

S1 Fig State-level associations between fatal police shooting rates and rate differences. (A) The White fatal police shooting rate and Black-White rate differences; (b) the White fatal police shooting rate and Hispanic-White rate differences; (c) Hispanic-White and Black-White rate differences. Data points are the posterior medians from Model 1. The median correlations across draws from the posteriors are in red text.
(PDF)

S2 Fig State-level associations between fatal police shooting rates and rate ratios. (A) The White fatal police shooting rate and Black-White rate ratios; (b) the White fatal police shooting rate and Hispanic-White rate ratios; (c) Hispanic-White and Black-White rate ratios. Data points are the posterior medians from Model 1. The median correlations across draws from the posteriors are in red text.
(PDF)

S3 Fig Race/ethnicity-state-level associations between police shooting deaths and household firearm ownership rates. Ribbons indicate 80% credible intervals. Rates are expressed as the number of police shootings per 100,000 over the 6-year study period. Cf. Fig 4, which uses a linear specification as opposed to the quadratic specification used here.
(PDF)

S4 Fig Rate ratios and rate differences versus household firearm ownership rates. Ribbons indicate 80% credible intervals. Rates are expressed as the number of police shootings per 100,000 over the 6-year study period.
(PDF)

S5 Fig Race/ethnicity-state-level associations between police shooting deaths and race/ethnicity-specific household firearm ownership rates. Ribbons indicate 80% credible intervals. Rates are expressed as the number of police shootings per 100,000 population over the 6-year study period. Cf. Fig 4, which uses overall firearm ownership rates at the state level, as opposed to this figure which uses non-Hispanic White versus other rates at the state level.
(PDF)

## References

[1] Zimring FE. When police kill. Cambridge, MA: Harvard University Press. 2017.

[2] Edwards F, Lee H, Esposito M. Risk of being killed by police use of force in the United States by age, race–ethnicity, and sex. Proceedings of the National Academy of Sciences. 2019;116(34):16793–8. 10.1073/pnas.1821204116 PMC6708348 31383756

[3] Sherman LW. Reducing Fatal Police Shootings as System Crashes: Research, Theory, and Practice. Annu Rev Criminol. 2018;1(1):421–49. doi: 10.1146/annurev-criminol-032317-092409

[4] Hemenway D, Azrael D, Conner A, Miller M. Variation in rates of fatal police shootings across US states: the role of firearm availability. J Urban Health. 2019;96(1):63–73. 30311055 10.1007/s11524-018-0313-z PMC6391295

[5] Nagin DS. Firearm Availability and Fatal Police Shootings. The ANNALS of the American Academy of Political and Social Science. 2020;687(1):49–57. doi: 10.1177/0002716219896259

[6] Sivaraman JJ, Marshall SW, Ranapurwala SI. State firearm laws, race and law enforcement–related deaths in 16 US states: 2010–2016. Injury Prevention. 2020;26(6):569–72. 32938691 10.1136/injuryprev-2020-043681

[7] Jain V, Hemenway D. Cross-State Relationship of Firearm Violence Between Police and Civilians: Gun Ownership as a Common Denominator. J Urban Health. 2024;101(5):951–4. doi: 10.1007/s11524-024-00904-5 39196466 PMC11461395

[8] Rogna M, Nguyen BD. Firearms law and fatal police shootings: a panel data analysis. Applied Economics. 2021;54(27):3121–37. doi: 10.1080/00036846.2021.2003290

[9] Kivisto AJ, Ray B, Phalen PL. Firearm Legislation and Fatal Police Shootings in the United States. Am J Public Health. 2017;107(7):1068–75. doi: 10.2105/AJPH.2017.303770 28520488 PMC5463213

[10] Doucette ML, Ward JA, McCourt AD, Webster D, Crifasi CK. Officer-involved shootings and concealed carry weapons permitting laws: analysis of gun violence archive data, 2014–2020. J Urban Health. 2022;99(3):373–84. 35536393 10.1007/s11524-022-00627-5 PMC9187822

[11] Crifasi CK, Ward J, McCourt AD, Webster D, Doucette ML. The association between permit-to-purchase laws and shootings by police. Inj Epidemiol. 2023;10(1):28. doi: 10.1186/s40621-023-00439-4 37386600 PMC10311703

[12] Correll J, Hudson SM, Guillermo S, Ma DS. The Police Officer’s Dilemma: A Decade of Research on Racial Bias in the Decision to Shoot. Social & Personality Psych. 2014;8(5):201–13. doi: 10.1111/spc3.12099

[13] Morral AR, Agniel D, Smart R, Schell TL. Household Firearm Ownership and Firearm Mortality. JAMA Netw Open. 2024;7(8):e2429335. doi: 10.1001/jamanetworkopen.2024.29335 39167407 PMC11339659

[14] Sheppard KG, Zimmerman GM, Fridel EE. Examining the Relevance of Contextual Gun Ownership on Fatal Police Shootings. Justice Quarterly. 2021;39(6):1214–36. doi: 10.1080/07418825.2021.1922733

[15] Murphy JP, Neil R, Paige JW. Methodological challenges for research on racial bias in police shootings. RAND Corporation. 2024. https://www.rand.org/pubs/research_reports/RRA243-8.html

[16] Ross CT. A Multi-Level Bayesian Analysis of Racial Bias in Police Shootings at the County-Level in the United States, 2011-2014. PLoS One. 2015;10(11):e0141854. doi: 10.1371/journal.pone.0141854 26540108 PMC4634878

[17] Ross CT, Winterhalder B, McElreath R. Racial Disparities in Police Use of Deadly Force Against Unarmed Individuals Persist After Appropriately Benchmarking Shooting Data on Violent Crime Rates. Social Psychological and Personality Science. 2020;12(3):323–32. doi: 10.1177/1948550620916071

[18] Klinger D, Rosenfeld R, Isom D, Deckard M. Race, Crime, and the Micro‐Ecology of Deadly Force. Criminology & Public Policy. 2015;15(1):193–222. doi: 10.1111/1745-9133.12174

[19] Nix J, Campbell BA, Byers EH, Alpert GP. A Bird’s Eye View of Civilians Killed by Police in 2015. Criminology & Public Policy. 2017;16(1):309–40. doi: 10.1111/1745-9133.12269

[20] Nix J, Shjarback JA. Factors associated with police shooting mortality: A focus on race and a plea for more comprehensive data. PLoS One. 2021;16(11):e0259024. doi: 10.1371/journal.pone.0259024 34758026 PMC8580236

[21] Schwartz GL, Jahn JL. Mapping fatal police violence across U.S. metropolitan areas: overall rates and racial/ethnic inequities, 2013-2017. PLOS ONE. 2020;15(6):e0229686. doi: 10.1371/journal.pone.0229686 PMC7313728 32579553

[22] Leslie TF, Frankenfeld CL, Hattery AJ. Differentiating Black and Hispanic: Outcome differences of segregated communities and police shootings in the USA, 2015–2020. Inj Epidemiol. 2022;9(1):8. 35241164 10.1186/s40621-022-00372-y PMC8892749

[23] Zare H, Meyerson NS, Delgado P, Crifasi C, Spencer M, Gaskin D. How place and race drive the numbers of fatal police shootings in the US: 2015–2020. Preventive Medicine. 2022;161:107132. doi: 10.1016/j.ypmed.2022.107132 35787843

[24] Cesario J, Johnson DJ, Terrill W. Is there evidence of racial disparity in police use of deadly force? Analyses of officer-involved fatal shootings in 2015–2016. Social Psychological and Personality Science. 2019;10(5):586–95.

[25] Tregle B, Nix J, Alpert GP. Disparity does not mean bias: Making sense of observed racial disparities in fatal officer-involved shootings with multiple benchmarks. In: Moule RK Jr., Fox B. Contemporary Issues in American Policing. New York, NY: Routledge. 2021.

[26] Fryer RG Jr. An Empirical Analysis of Racial Differences in Police Use of Force. Journal of Political Economy. 2019;127(3):1210–61. doi: 10.1086/701423

[27] Siegel M. Racial disparities in fatal police shootings: An empirical analysis informed by critical race theory. Boston University Law Review. 2020;100:1069–92.

[28] Mesic A, Franklin L, Cansever A, Potter F, Sharma A, Knopov A, et al. The Relationship Between Structural Racism and Black-White Disparities in Fatal Police Shootings at the State Level. J Natl Med Assoc. 2018;110(2):106–16. doi: 10.1016/j.jnma.2017.12.002 29580443

[29] Police shootings database 2015-2024: Search by race, age, department. Washington Post. 2024. https://www.washingtonpost.com/graphics/investigations/police-shootings-database/

[30] Centers for Disease Control and Prevention. Single-Race Population Estimates. 2025. https://wonder.cdc.gov/single-race-population.html

[31] Schell TL, Peterson S, Vegetabile BG, Scherling A, Smart R, Morral AR. State-level estimates of household firearm ownership. RAND Corporation. 2020. https://www.rand.org/pubs/tools/TL354.html

[32] Gelman A, Fagan J, Kiss A. An Analysis of the New York City Police Department’s “Stop-and-Frisk” Policy in the Context of Claims of Racial Bias. Journal of the American Statistical Association. 2007;102(479):813–23. doi: 10.1198/016214506000001040

[33] Gelman A, Carlin JB, Stern HS, Dunson DB, Vehtari A, Rubin DB. Bayesian data analysis. Third Edition ed. Boca Raton, FL: CRC Press. 2013.

[34] Bürkner PC. brms: An R package for Bayesian multilevel models using Stan. J Stat Soft. 2017;80(1).

[35] Morral AR, Smart R, Schell TL, Vegetabile B, Thomas E. Geographic and demographic differences in the proportion of individuals living in households with a firearm, 1990-2018. JAMA Netw Open. 2024;7(2):e240562. doi: 10.1001/jamanetworkopen.2024.0562 PMC10902733 38416496

[36] Ward JA, Cepeda J, Jackson DB, Johnson O, Webster DW, Crifasi CK. National burden of injury and deaths from shootings by police in the United States, 2015‒2020. Am J Public Health. 2024;114(4):387–97. 38478866 10.2105/AJPH.2023.307560 PMC10937603

[37] Nix J. On the challenges associated with the study of police use of deadly force in the United States: A response to Schwartz & Jahn. PLoS One. 2020;15(7):e0236158. doi: 10.1371/journal.pone.0236158 32722714 PMC7386827

[38] Ward JA, Johnson O, Cepeda JA, Jackson DB, Webster DW, Crifasi CK. Social and policy characteristics associated with injurious shootings by police in US counties: A multilevel analysis, 2015–2020. Social Science & Medicine. 2024;362:117460. 39488173 10.1016/j.socscimed.2024.117460

