# Supplementary material for: Racial and ethnic disparities in fatal police shootings: Variation across U.S. states and the role of firearm ownership
Source: PLoS One. 2026 Mar 11;21(3):e0333424. doi: 10.1371/journal.pone.0333424 (PMC12978442; doi:10.1371/journal.pone.0333424)
Supplement: S1 Table — Coefficients are expressed as log rate ratios, brackets indicate 80% Bayesian credible intervals. Both models also contain the log of population as an offset, random intercepts for year and state, and state-race/ethnicity random slopes. Cf. S4 Table, which contains the regression output for the models used in the main results. N = 900. (PDF) [file pone.0333424.s001.pdf]

S1 Table. Output for negative binomial models predicting fatal police shootings using imputed data

	**Model 1b**	**Model 2b**
Intercept	-12.896 (-13.018, -12.777)	-14.132 (-14.431, -13.839)
Black	1.052 (0.944, 1.156)	1.714 (1.400, 2.034)
Hispanic	-0.093 (-0.214, 0.018)	0.511 (0.187, 0.845)
Firearm Ownership		3.294 (2.557, 4.014)
*Black * Firearm Ownership*		-1.777 (-2.599, -0.936)
*Hispanic * Firearm Ownership*		-1.803 (-2.719, -0.921)
LOOIC	3346.2	3339.1

Coefficients are expressed as log rate ratios, brackets indicate 80% Bayesian credible intervals. Both models also contain the log of population as an offset, random intercepts for year and state, and state-race/ethnicity random slopes. Cf. Table S4, which contains the regression output for the models used in the main results. N = 900.
