# Supplementary material for: Racial and ethnic disparities in fatal police shootings: Variation across U.S. states and the role of firearm ownership
Source: PLoS One. 2026 Mar 11;21(3):e0333424. doi: 10.1371/journal.pone.0333424 (PMC12978442; doi:10.1371/journal.pone.0333424)
Supplement: S2 Table — (PDF) [file pone.0333424.s002.pdf]

S2 Table. Posterior predictive checks

Model	Mean of Y	Maximum of Y	Proportion of Y that is Zero	Variance of Y
1	0.50	0.29	0.51	0.54
2	0.52	0.36	0.71	0.52

Each row of Table S2 presents results for one model. The second through fifth columns show posterior predictive checks (PPCs). For the PPCs, the values reflect the proportion of simulations from the posterior in which the statistic was greater than or equal to that from the observed data. Values near 0.5 mean that a model is capturing that aspect of the distribution of the outcome (Y) well. Table S2 indicates both models fit the data well, albeit imperfectly. For instance, in 71% of simulations the proportion of state-year-race/ethnicities with no shootings (i.e., Y = 0) was higher than (or equal to) the observed proportion of 0.28.
