# Supplementary material for: Racial and ethnic disparities in fatal police shootings: Variation across U.S. states and the role of firearm ownership
Source: PLoS One. 2026 Mar 11;21(3):e0333424. doi: 10.1371/journal.pone.0333424 (PMC12978442; doi:10.1371/journal.pone.0333424)
Supplement: S3 Table — Fatal police shooting rates are per 100,000 residents over the 6-year study period. 80% Bayesian credible intervals are in brackets. Cf. Table 1, which contains the analogous quantities using the observed data as opposed to these model-based estimates. (PDF) [file pone.0333424.s003.pdf]

S3 Table. Model-based fatal police shooting rates by race and national disparities

Race/Ethnicity	Fatal Police Shooting Rate	Risk Difference Compared to White Rate	Risk Ratio Compared to White Rate
**Panel A: Estimates from Model 1**			
White	1.41 (1.35, 1.46)	N/A	N/A
Black	3.54 (3.36, 3.73)	2.14 (1.95, 2.33)	2.52 (2.36, 2.68)
Hispanic	1.73 (1.62, 1.85)	0.33 (0.20, 0.45)	1.23 (1.14, 1.33)
**Panel B: Estimates from Model 2**			
White	1.40 (1.35, 1.46)	N/A	N/A
Black	3.55 (3.37, 3.73)	2.14 (1.96, 2.33)	2.53 (2.37, 2.7)
Hispanic	1.74 (1.63, 1.86)	0.34 (0.21, 0.46)	1.24 (1.15, 1.34)

Fatal police shooting rates are per 100,000 residents over the 6-year study period. 80% Bayesian credible intervals are in brackets. Cf. Table 1, which contains the analogous quantities using the observed data as opposed to these model-based estimates.
