# Supplementary material for: Racial and ethnic disparities in fatal police shootings: Variation across U.S. states and the role of firearm ownership
Source: PLoS One. 2026 Mar 11;21(3):e0333424. doi: 10.1371/journal.pone.0333424 (PMC12978442; doi:10.1371/journal.pone.0333424)
Supplement: S4 Table — Coefficients are expressed as log rate ratios, brackets indicate 80% Bayesian credible intervals. Both models also contain the log of population as an offset, random intercepts for year and state, and state-race/ethnicity random slopes. N = 900. (PDF) [file pone.0333424.s004.pdf]

S4 Table. Output for negative binomial models predicting fatal police shootings

	Model 1	Model 2
Intercept	-12.974 (-13.099, -12.849)	-14.228 (-14.514, -13.945)
Black	1.069 (0.959, 1.180)	1.804 (1.463, 2.111)
Hispanic	-0.082 (-0.209, 0.032)	0.602 (0.265, 0.950)
Firearm Ownership		3.328 (2.617, 4.013)
*Black * Firearm Ownership*		-1.959 (-2.772, -1.081)
*Hispanic * Firearm Ownership*		-2.086 (-3.059, -1.153)
LOOIC	3258.5	3247.2

Coefficients are expressed as log rate ratios, brackets indicate 80% Bayesian credible intervals. Both models also contain the log of population as an offset, random intercepts for year and state, and state-race/ethnicity random slopes. N = 900.
