# Supplementary material for: Racial and ethnic disparities in fatal police shootings: Variation across U.S. states and the role of firearm ownership
Source: PLoS One. 2026 Mar 11;21(3):e0333424. doi: 10.1371/journal.pone.0333424 (PMC12978442; doi:10.1371/journal.pone.0333424)
Supplement: S5 Table — Coefficients are expressed as log rate ratios, brackets indicate 80% Bayesian credible intervals. Both models also contain the log of population as an offset, random intercepts for year and state, and state-race/ethnicity random slopes. N = 900. (PDF) [file pone.0333424.s005.pdf]

S5 Table. Output for Poisson models predicting fatal police shootings

	**Model 1**	**Model 2**
Intercept	-12.983 (-13.103, -12.862)	-14.233 (-14.514, -13.946)
Black	1.074 (0.962, 1.183)	1.801 (1.478, 2.108)
Hispanic	-0.082 (-0.210, 0.034)	0.599 (0.266, 0.963)
Firearm Ownership		3.342 (2.628, 4.025)
*Black * Firearm Ownership*		-1.949 (-2.745, -1.120)
*Hispanic * Firearm Ownership*		-2.07 (-3.062, -1.146)
LOOIC	3264.1	3251.0

Coefficients are expressed as log rate ratios, brackets indicate 80% Bayesian credible intervals. Both models also contain the log of population as an offset, random intercepts for year and state, and state-race/ethnicity random slopes. N = 900.
