# Supplementary material for: Racial and ethnic disparities in fatal police shootings: Variation across U.S. states and the role of firearm ownership
Source: PLoS One. 2026 Mar 11;21(3):e0333424. doi: 10.1371/journal.pone.0333424 (PMC12978442; doi:10.1371/journal.pone.0333424)
Supplement: S2 Fig — (A) The White fatal police shooting rate and Black-White rate ratios; (b) the White fatal police shooting rate and Hispanic-White rate ratios; (c) Hispanic-White and Black-White rate ratios. Data points are the posterior medians from Model 1. The median correlations across draws from the posteriors are in red text. (PDF) [file pone.0333424.s007.pdf]

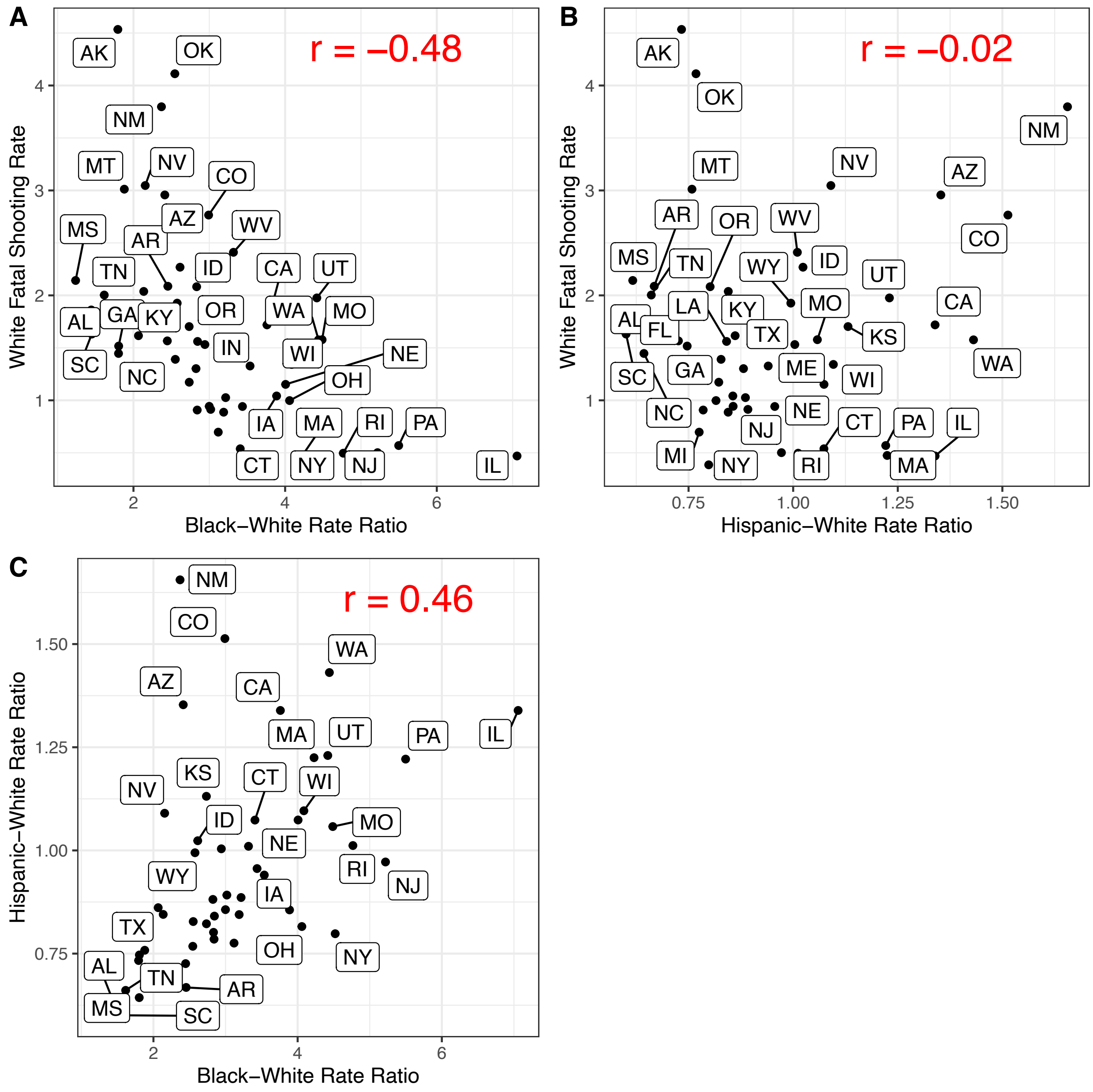

S2 Fig. State-level associations between fatal police shooting rates and rate ratios. (A) The White fatal police shooting rate and Black-White rate ratios; (b) the White fatal police shooting rate and Hispanic-White rate ratios; (c) Hispanic-White and Black-White rate ratios. Data points are the posterior medians from Model 1. The median correlations across draws from the posteriors are in red text.
