# Supplementary material for: Racial and ethnic disparities in fatal police shootings: Variation across U.S. states and the role of firearm ownership
Source: PLoS One. 2026 Mar 11;21(3):e0333424. doi: 10.1371/journal.pone.0333424 (PMC12978442; doi:10.1371/journal.pone.0333424)
Supplement: S4 Fig — Ribbons indicate 80% credible intervals. Rates are expressed as the number of police shootings per 100,000 over the 6-year study period. (PDF) [file pone.0333424.s009.pdf]

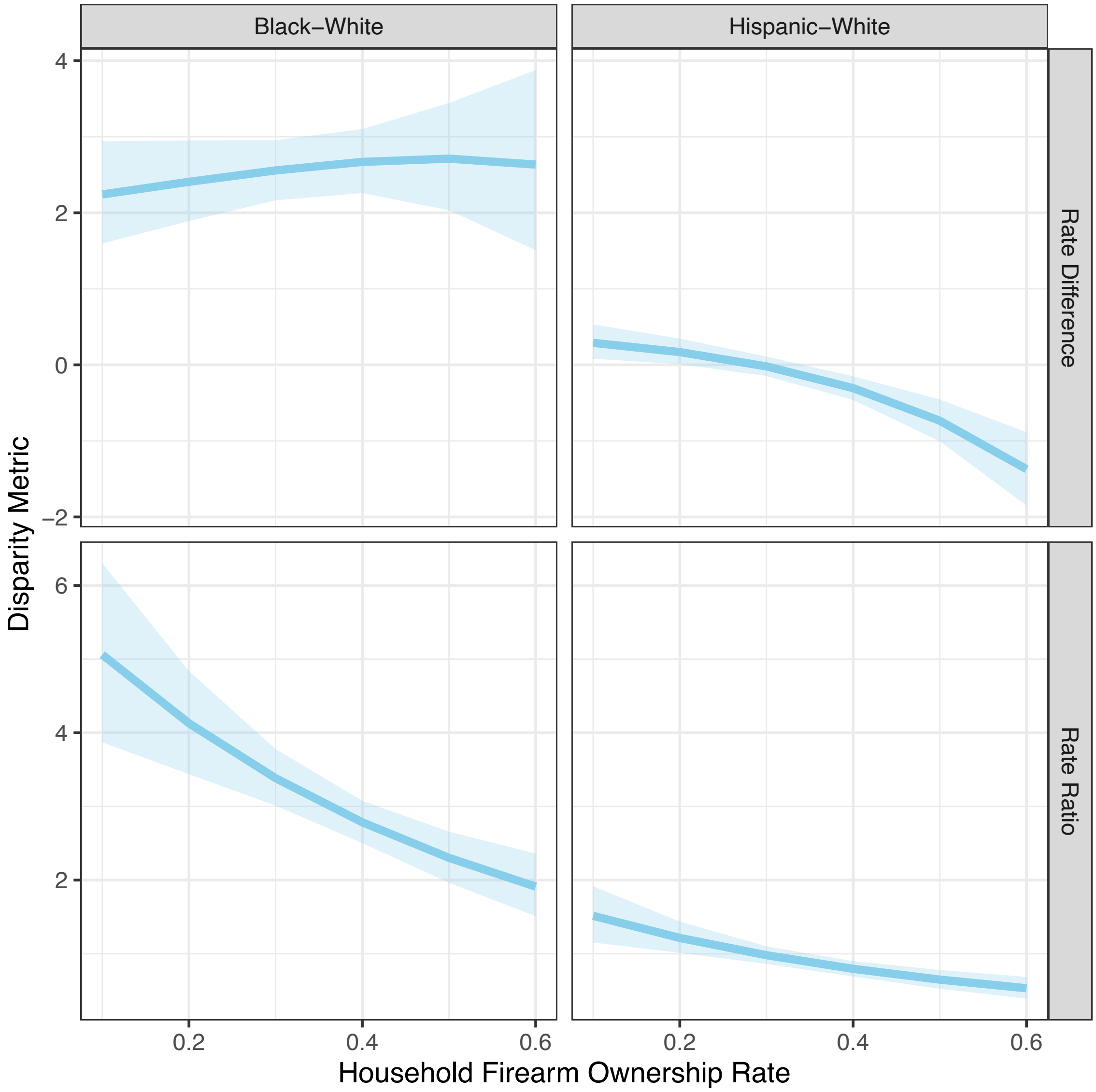

S4 Fig. Rate ratios and rate differences versus household firearm ownership rates. Ribbons indicate 80% credible intervals. Rates are expressed as the number of police shootings per 100,000 over the 6-year study period.
